# Transmission and genetic diversity of *Enterococcus faecalis *among layer chickens during hatch

**DOI:** 10.1186/1751-0147-53-56

**Published:** 2011-10-23

**Authors:** Mette E Fertner, Rikke H Olsen, Magne Bisgaard, Henrik Christensen

**Affiliations:** 1 Department of Veterinary Disease Biology, Faculty of Life Sciences, University of Copenhagen, 4 Stigbøjlen, DK-1870 Frederiksberg C, Denmark

## Abstract

**Background:**

Studies on transmission of *Enterococcus faecalis *among chickens during hatch have not been carried out so far. Information about vertical transmission and subsequent spreading and colonization of the cloacal mucosa through cloacal 'drinking' during hatch are important to understand the epidemiology of *E. faecalis *infections. In the present investigation vertical transmission and subsequent spreading and colonization of the cloacal mucosa of chickens by *E. faecalis *through cloacal 'drinking' were examined.

**Methods:**

Two different batches of layer chickens originating from 45 weeks old Brown and White Lohmann parents, respectively from the same farm were sampled in the hatcher. Isolates were confirmed to be *E. faecalis *by polymerase chain reaction (PCR) and further by multilocus sequence typing (MLST) to state their population structure and comparison made to sequence types previously obtained from chicken.

**Results:**

A total of 480 chickens were swabbed from the cloacae just after hatch and after 24 hours. A total of 101 isolates were confirmed as *E. faecalis *by a species specific PCR. The prevalence of *E. faecalis *increased from 14% at 0 h to 97% after 24 h for the Brown Lohmann chickens and from 0.5% to 23% for the White Lohmann flock. The 84 isolates analysed by MLST were distributed on 14 sequence types (ST). Three ST (401, 82 and 249) accounted for 64% of all isolates analysed by MLST after 24 h. ST 82 has previously been reported from amyloid arthropathy and other lesions in poultry.

**Conclusions:**

The present findings demonstrated a high potential of a few contaminated eggs or embryos to rapidly facilitate the spread of *E. faecalis *to almost all chickens during hatch.

## Background

*Enterococcus faecalis *is part of the normal intestinal microbial flora of poultry and man [1]. Under most conditions, *E. faecalis *is considered as an opportunistic pathogen. In humans, *E. faecalis *represents one of the most important causes of nosocomial infections [2]. Clinical conditions observed in poultry include growth depression [3], pulmonary hypertension syndrome [4], and amyloid arthropathy [5] in addition to first week mortality [6]. In broiler parents, 12 different sequence types (STs) were reported from valvular endocarditis, septicaemia, salpingitis, peritonitis, arthritis and combinations of some of these conditions [6]. A specific clone of *E. faecalis *has been associated with amyloid arthropathy [7] and was subsequently identified as ST 82 [8]. However, most STs of *E. faecalis *seem to have the potential to induce amyloidosis and chronic infections characteristic for *E. faecalis *seem essential for developing amyloidosis [6]. Unfortunately, knowledge on epidemiology and pathogenesis of *E. faecalis *infections in poultry has remained fragmentary. This has limited implementation of preventive strategies despite of recent observations suggesting that *E. faecalis *represents a new zoonosis [9].

Several experimental investigations have been employed to demonstrate possible routes of transmission of *E. faecalis *among chickens [10-13]. To the knowledge of the authors, studies on transmission of *E. faecalis *among chicks during hatch have not been carried out so far. For the same reason, vertical transmission and subsequent spreading and colonization of the cloacal mucosa of chickens through cloacal 'drinking' were examined during hatch. In addition, isolates of *E. faecalis *were sequence typed and compared with STs previously obtained from clinical conditions in poultry.

## Methods

### Flock data

Two studies were carried out on clinically healthy chicks from a Danish hatchery. Chickens investigated originated from two Brown and White Lohmann breeder flocks from the same farm, aged 45 weeks (Table 1). Upon arrival at the hatchery, all eggs were formalin fumigated before incubation. Hatching started on day 20 of incubation. Two hatchers, each of which contained 8,000 eggs, were randomly selected on day 20 (time 0 h) just as newly hatched chickens were randomly selected from trays covering the whole hatcher (Table 1). The same procedure was applied for selection of chickens on day 21 of incubation (time 24 h) (Table 1). Due to an expected lower prevalence of *E. faecalis *a higher number of chickens was swabbed on day 20.

**Table 1:** Prevalence of *Enterococcus faecalis *originating from Brown or White Lohmann layer parents flocks aged 45 weeks from the same farm.

Flock	Age of chickens (hours)	PCR positive *E. faecalis*	Total samples	Prevalence in %	CI^95% ^*	Yates corrected Chi^2 ^value	*P*
Brown	0	14	100	14.0	0.094-0.247	98.8	0.000
Brown	24	58	60	96.7	0.991-0.999		
White	0	1	200	0.5	0.001-0.036	41.4	0.000
White	24	28	120	23.3	0.161-0.319		

All isolates were confirmed to be *E. faecalis *by a species specific polymerase chain reaction (PCR).

* CI^95% ^is the 95% confidence interval.

### Sampling and bacterial isolation

A total of 300 newly hatched chicks were sampled at 0 h while 180 chickens were sampled at 24 h. A cloacal swab sample from each chick investigated was collected with a sterile cotton wool swab rotated in contact with the cloacal mucosa. The swab was plated onto a blood agar plate (BA, Blood Agar Base, CM55, Oxoid, Basingstoke, UK) with 5% sterile calf blood. The same swab was subsequently used to inoculate a 0.01% potassium tellurite agar plate (Merck, Whitehouse Station, USA). All agar plates were incubated at 37°C for 24 h after which the qualitative composition of the microflora on BA was evaluated based upon colony morphology. From plates demonstrating colonies characteristic of *E. faecalis *a single colony was subcultured and stored at -80°C in hearth infusion broth (HIB, Difco, Brøndby, Denmark) added 15% glycerol. In addition, randomly selected isolates with colonies typical of *Staphylococcus hyicus *were subjected 16S rRNA gene sequencing for verification.

### Analysis of *E. faecalis*

Boiling lysates from all isolates were used in an initial *E. faecalis *specific polymerase chain reaction (PCR) amplifying the ddl*_E. faecalis _*of 941 base pairs by means of the primers E_1 _5'-ATCAAGTACAGTTAGTCTT-3' (TAG Copenhagen, Denmark) and E_2 _5'-ACGATTCAAAGCTAACTG-3' (TAG Copenhagen) [14]. Volumes of 50 μl were set up under the following PCR-conditions: 94°C for 2 min of initial denaturation, 30 cycles of 94°C for 1 min of denaturation, 54°C for 1 min of annealing and 72°C for 1 min of amplification; followed by a final extension of 72°C for 10 min. PCR products were run in a 1% agarose gel (Lonza, Rockland ME, USA) and stained with ethidium bromide (0.07%, Sigma).

Eighty four isolates identified as *E. faecalis *in the specific PCR were further characterized by a combination of the single nucleotide polymorphism (SNP) method [15] specific for ST 82 of *E. faecalis *and multilocus sequence typing (MLST). The 84 isolates represented all four samplings (Table 1). Unfortunately 17 out of 101 isolates identified as *E. faecalis *by PCR were not available for investigation. They represented four samples from 0 h and 13 from 24 h sampling times of the Brown Lohmann chickens with similar properties to isolates investigated so that their loss did not bias the investigation. For both methods DNA was first purified according to the manufacturer's instructions for DNA purification of Gram positive bacteria (DNeasy Blood & Tissue Kit from Quiagen, Hilden, Germany). Extracted DNA was run in a Real-Time PCR (RT-PCR) for SNP screening for the clone ST 82 of *E. faecalis *[15]. Six primers were used to amplify one out of two possible allelic sequences in each of the two genes, *pstS *and *xpt *[15]. Reactions were setup in volumes of 25 μl and run in a MxPro3000 (Strategene) using the following PCR conditions: 95°C for 10 min of initial denaturation, and 40 cycles of 95°C for 30 sec of denaturation, 58°C for 1 min of annealing, 72°C for 15 sec of elongation and terminated by 1 cycle of dissociation analysis consisting of 95°C for 1 min of denaturation, 55°C for 30 sec of annealing and 95°C for 30 sec of denaturation [15].

Isolates not identified as ST 82 and eight isolates found positive by the RT-PCR (controls) were analysed by MLST by amplifying fragments of seven housekeeping genes. Primers and PCR conditions used were performed as stated on the MLST homepage for *E. faecalis *[16,17]. Amplified products were purified and sequenced by Macrogen (Seoul, Korea). Products were sequenced in both directions and compared with published alleles using the computer program CLC bio (Aarhus, Denmark). Based on the seven allelic numbers, a ST was assigned to each isolate.

To analyse the evolutionary relationships between STs on sequence level, the seven sequences for the alleles of each ST were concatenated and a multiple alignment created by ClustalX [18]. In the same program neighbor joining analysis was carried out, and the phylogenetic tree was constructed from MEGA4 [19]. 16S rRNA gene sequencing of two isolates was performed according to previous reports [20,21]. The isolates were selected to represent a haemolytic and non-haemolytic variant of the most frequent colony morphology not representing *Escherichia coli *and *E. faecalis *and found negative in the *E. faecalis *species specific PCR.

## Results

One hundred and one isolates were identified as *E. faecalis *by the species specific PCR (Table 1). The prevalence increased from 14% at 0 h to 97% after 24 h among chickens originating from Lohmann Brown breeders while the prevalence increased from 0.5% to 23% among offspring from Lohmann White layers after 24 h. On both occasions, the increase was significant (Table 1).

Fourteen STs were detected by MLST (Table 2). Detection of ST 82 by RT-PCR was confirmed by MLST for all control isolates. As to chickens originating from the Lohmann Brown flock, the number of STs increased from four at time 0 to nine after 24 h, while the same figures for the Lohmann White were one and eight, respectively (Table 2). The genetic diversity did not change significantly from 0 to 24 h. Chickens originating from the two different parent flocks only shared four out the 14 STs (82, 249, 273, 314) although both flocks originated from the same farm.

**Table 2:** Distribution of sequence types (ST) isolated from chickens hatched from Brown or White Lohmann layer parents sampled at 0 or 24 hour in the hatcher.

				*Enterococcus faecalis*		
					
Flock	Sampling time (hour)	Number of isolates	ST	Massive growth in pure culture on primary plates	Poor growth in pure culture or mixed growth on primary plates	Total prevalence in % *
Brown	0	5	82	1	4	5.0
Brown	0	2	249	1	1	2.0
Brown	0	2	314	2	0	2.0
Brown	0	1	401	0	1	1.0
Brown	24	1	4	0	1	1.7
Brown	24	14	82	1	13	23.3
Brown	24	3	141	1	2	5.0
Brown	24	16	249	3	13	26.7
Brown	24	1	273	0	1	1.7
Brown	24	1	314	0	1	1.7
Brown	24	1	402	0	1	1.7
Brown	24	1	400	0	1	1.7
Brown	24	5	401	0	5	8.3
White	0	1	177	0	1	0.5
White	24	2	32	1	1	1.7
White	24	9	82	4	5	7.5
White	24	1	100	1	0	0.8
White	24	5	174	2	3	4.2
White	24	3	228	2	1	2.5
White	24	3	273	0	3	2.5
White	24	5	314	1	4	4.2
White	24	2	249	1	1	1.7

* with respect to all 101 isolates confirmed by polymerase chain reaction (PCR) to be *E. faecalis*

**Figure 1:**
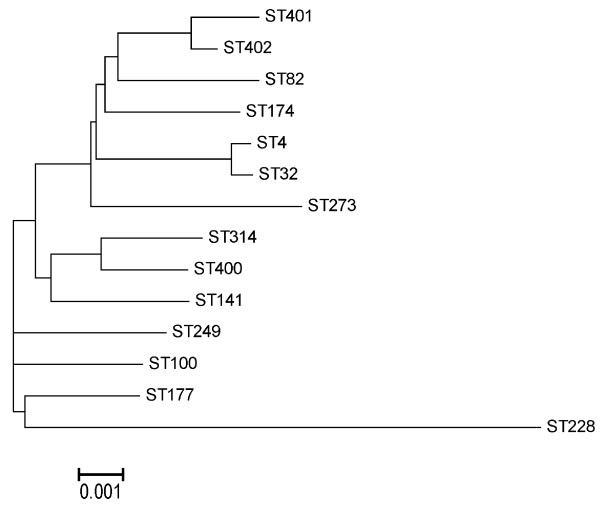
**Phylogeny of sequence types (ST) of *Enterococcus faecalis *isolated from hatched chickens during the first 24 h of life based on neighbour joining analysis of concatenated sequences of the seven house-keeping genes used for multilocus sequence typing (MLST)**.

For Lohmann Brown, ST 82 increased from 5.0% to 23.3% during the 24 hours of hatch (Table 2). For ST 82 at 0 h, one of the five cultures demonstrated massive growth in pure culture of *E. faecalis*, indicating vertical transmission while four chickens demonstrated poor growth of *E. faecalis *(Table 2). After 24 h only one out of 14 isolates of ST 82 showed massive growth in pure culture, whereas the rest were obtained from a mixed culture of *E. coli *and *E. faecalis*. For ST 249 the same tendency was observed as for ST 82: the prevalence increased from 2.0% to 26.7% from 0 to 24 h. After 24 h three out of 16 plates demonstrated massive growth in pure culture of *E. faecalis*. For the other STs, most isolates after 24 h were obtained from plates with poor growth or in mixed culture. For Lohmann White, only a single isolate in mixed culture was found at 0 h. This isolate belonged to ST 177. After 24 h, most STs were represented by isolates that were both obtained from plates demonstrating either massive growth in pure culture or from plates with poor growth or in mixed culture. The phylogenetic analysis of the concatenated sequences showed the most distant relationship of ST 228 (Figure 1). ST 4 and ST 32 only diverged in one (*pstS*) of the seven genes (99.9% DNA similarity) and ST 401 and ST 402 in another *yqi *(99.8% DNA similarity) whereas none of the other STs were closely related (< 99.6% DNA similarity). Two isolates (4-19, 4-24) selected to represent bacteria frequently isolated that did not belong to *E. faecalis *were 16S rRNA sequenced. They showed similarities of 99.7% and 99.6% to the type strain of *S. hyicus*, respectively. This colony type was often observed in a mixed microflora also including *E. faecalis *and *E. coli*. In five cases, abundant growth in almost pure culture of *S. hyicus *(more than 90% of colonies) was observed indicating vertical transmission of this bacterium.

## Discussion

During the first three days of life, the intestine of chicks becomes colonized with bacteria [22-24]. *E. faecalis *and *Enterococcus faecium *constitute the enterococcal species most commonly isolated from day-old chicks [24]. Although the enterococcal distribution changes with age [24,25], *E. faecalis *has also been found in the intestine of adult poultry [1]. The quality of day-old chickens have major impact on first week mortality and we have recently shown that *E. coli *and *E. faecalis *were the most significant bacterial pathogens associated with first week mortality [25].

Our results show that *E. faecalis *colonized the cloacal mucosa of chickens during hatch. A significant increase in the prevalence of *E. faecalis *of seven and 46 times within the two flocks investigated, respectively, can be explained as a result of horizontal transmission through the oral route and by cloacal 'drinking' between chicks in the hatcher. Both oral uptake and cloacal 'drinking' are likely routes for bacterial transmission in naive chickens. The latter route was documented by van der Sluis *et al. *[26], who showed that labelled material taken up by the cloaca could later be found in the bursa of Fabricius, caecum and small intestine of the same chickens. Horizontal transfer of bacteria can occur by contact between chickens, contact with contaminated egg shells as well as air-borne transmission of bacteria through hatchery fluff [23,27].

In the present investigation we have assumed that a ST represents a clonal entity of *E. faecalis *in relation to colonization and transmission. It is further assumed that massive growth of *E. faecalis *in pure culture at 0 and 24 h represents vertical transmission from infected eggs. Poor growth in pure culture of *E. faecalis *and poor/good growth of *E. faecalis *on primary plates with other bacteria at 0 and 24 h might originate from shells and represent contaminated eggs or horizontal transmission in the hatcher. For only one ST (314) with two isolates observed at 0 h in Lohmann Brown, a vertical transmission was indicated while only five out of the 43 isolates that were MLST typed at 24 h indicated vertical transmission. For the Lohmann White, a low prevalence at 0 h and lack of transmission of the same ST (177) after 24 h indicated low or no vertical transmission of this ST while vertical transmission of 12 isolates were indicated after 24 h. For isolates at 24 h, 23 and five isolates out of the 73 belonged to ST 82 and ST 174, respectively. These STs and ST 177 made up 81% of the isolates associated with lesions in broiler breeders underlining their disease potential which might have induced a late hatch [6]. ST 177 was only detected once and only at 0 h in a mixed microflora indicating horizontal transmission or infection as a result of shell contamination. ST 32 has only rarely been isolated from lesions in broiler breeders [6] while ST 149 has not previously been isolated from lesions in chickens but from human infections [16]. Seven of the 14 STs (4, 32, 82, 117, 141, 174, and 249) have previously been reported from poultry whereas three STs (400, 401, and 402) were demonstrated for the first time. The three STs that demonstrated the highest prevalences were either unique to this investigation (401), common in chicken (82) or reported from chicken faeces in Norway (249) [16]. ST 82 has mainly been isolated from amyloid arthropathy in layers [8] and from different lesions in broiler parent breeders, including amyloidosis and arthritis [6]. This ST has also been reported from human clinical source [16].

Some 25% of the isolates demonstrated abundant growth in pure culture supporting a genuine vertical transmission of *E. faecalis*. Such route has previously only been indicated to play a minor role during infection of chickens with the clone of *E. faecalis *involved in amyloid arthropathy [11]. Whether this observation represent both genuine vertical infections as a result of *in ovo *infection or shell contamination from the cloacal mucosa of the hen or environment and subsequent shell penetration remains to be investigated. Chickens hatched from eggs dipped in fluid with salmonella solution were found to transfer salmonella to the gut of 44% of chickens hatched from salmonella free eggs [23]. Similar data are not available for *E. faecalis *but it is assumed that lack of proper egg hygiene including the use of floor-and dirty eggs, washed eggs and eggs demonstrating almost invisible cracks represents a major risk for transmission of *E. faecalis*. Unfortunately the conditions under which certain STs result in disease and productions loss have not been clearly established and further investigations are needed for clarification. The relative high occurrence of *S*. *hyicus *and its impact on subsequent flock health needs further investigations since only limited data have been published on this bacterium from chickens [28,29]. Our investigation only reported a snapshot of bacterial transfer between chickens in the hatcher. Further investigations are needed to investigate the impact of the present findings.

## Conclusions

The present findings demonstrated a high potential of a few contaminated eggs or embryos to rapidly spread *E. faecalis *infection to almost all chickens during hatch.

## Competing interests

The authors declare that they have no competing interests.

## Authors' contributions

All authors took part in sampling of isolates. MEF mainly carried out the initial characterization of isolates and further molecular genetic studies assisted by RHO and MB. HC participated in the sequence analysis. MEF drafted the manuscript assisted by HC, MB and RHO. All authors read and approved the final manuscript.
